# Cellular and molecular mechanisms associated with ischemic stroke severity in female mice with chronic kidney disease

**DOI:** 10.1038/s41598-019-42933-0

**Published:** 2019-04-23

**Authors:** Lucie Hénaut, Maria Grissi, François Brazier, Maryam Assem, Sabrina Poirot-Leclercq, Gaëlle Lenglet, Cédric Boudot, Carine Avondo, Agnès Boullier, Gabriel Choukroun, Ziad. A Massy, Saïd Kamel, Jean-Marc Chillon

**Affiliations:** 1 MP3CV, EA7517, CURS, Jules Verne University of Picardie, Amiens, 80025 France; 2 Division of Nephrology, Amiens University Hospital, Amiens, 80054 France; 3 Division of Nephrology, Ambroise Paré University Hospital, Boulogne-Billancourt, 92104 France; 4 Inserm U1018, CESP Team 5, UVSQ, Villejuif, 94807 France; 5 Laboratory of Biochemistry, Amiens University Hospital, Amiens, 80054 France; 6 DRCI, Amiens University Hospital, Amiens, 80054 France

**Keywords:** Chronic kidney disease, Stroke

## Abstract

Ischemic stroke is highly prevalent in chronic kidney disease (CKD) patients and has been associated with a higher risk of neurological deterioration and in-hospital mortality. To date, little is known about the processes by which CKD worsens ischemic stroke. This work aimed to investigate the cellular and molecular mechanism associated with ischemic stroke severity in an *in vivo* model of CKD. CKD was induced through right kidney cortical electrocautery in 8-week-old female C57BL/6 J mice followed by left total nephrectomy. Transient middle cerebral artery occlusion (tMCAO) was performed 6 weeks after left nephrectomy. Twenty-four hours after tMCAO, the infarct volumes were significantly wider in CKD than in SHAM mice. CKD mice displayed decreased neuroscore, impaired ability to remain on rotarod device, weaker muscular strength and decreased prehensile score. Apoptosis, neuronal loss, glial cells recruitment and microglia/macrophages M_1_ signature genes CD32, CD86, IL-1β, IL-6, MCP1 and iNOS were significantly increased within ischemic lesions of CKD mice. This effect was associated with decreased AMP kinase phosphorylation and increased activation of the NFΚB pathway. Pharmacological targeting of AMP kinase activity, which is known to block microglia/macrophages M_1_ polarization, appears promising to improve stroke recovery in CKD.

## Introduction

Stroke is the third cause of cardiovascular death in patients suffering from chronic kidney disease (CKD)^1^. The ischemic subtype of stroke accounts for approximately 75% of the overall strokes experienced by dialysis patients^1–3^ and advanced CKD has been associated with a higher risk of neurological deterioration, in-hospital mortality and poor functional outcomes following acute ischemic stroke^4^.

The high frequency of stroke observed in CKD patients can be explained by the strong prevalence of traditional cardiovascular risk factors such as hypertension, diabetes, inflammation, and dyslipidemia. In addition, other non-traditional risk factors related to kidney injury may predispose CKD patients to either ischemic or hemorrhagic strokes. These non-traditional risk factors include the CKD-associated disorders of bone and mineral metabolism (CKD-MBD), oxidative stress, endothelial dysfunction and the accumulation of uremic toxins (UTs). CKD was also reported to increase atrial fibrillation inducibility through increased atrial structural remodeling^5^, which subsequently increases the risk of stroke occurrence^6^. Therefore, the risk of stroke increases linearly and additively with declining GFR and increasing proteinuria^3,7^.

To date, little is known about the processes by which CKD worsens ischemic stroke. In addition, it has to be noted that most of our current knowledge in the field is based on data obtained from *in vitro* studies^8^. In a previous work, Yates *et al*. reported that acute kidney injury had no influence on infarct size or neurologic function in male wistar rats subjected to transient middle cerebral artery occlusion (tMCAO)^9^. However, since then no further studies have been undertaken to assess the influence of CKD on stroke recovery in rodents. Therefore, the development of *in vivo* approaches allowing identifying underlying causes and successful medical treatments remains a major challenge. In the present study, we sought to investigate the cellular and molecular mechanisms by which uremia worsened the severity of ischemic stroke after tMCAO in mice. To our knowledge, this report is the first to evaluate the impact of CKD on the nature of ischemic brain lesions and subsequent cognitive impairments in an *in vivo* model.

## Methods

### Animals and diet

**Figure 1:**
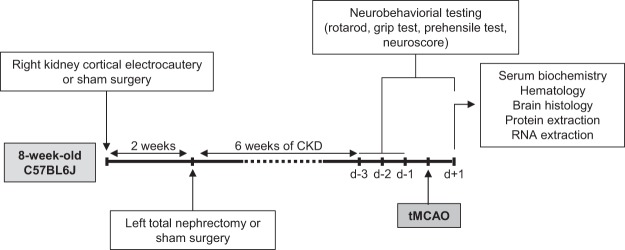
Schematic illustration of the experimental protocol. Abbreviations: CKD: chronic kidney disease, tMCAO: transient middle cerebral artery occlusion.

All experiments were performed on female C57BL/6 J mice purchased from Charles River Laboratories (Lyon, France). Animals were housed in polycarbonate cages in temperature- and humidity-controlled rooms with a 12-hour/12-hour light/dark cycle and were given standard chow (Harlan Teklad Global Diet 2018, Harlan, Bicester, UK) and tap water *ad libitum*. Animals were handled in accordance with the French legislation. The protocol was approved by an institutional animal care committee (*Comité Régional d’Ethique en Matière d’Expérimentation Animale de Picardie*, Amiens, France) and the French Ministry of Education and Research (Protocol ID: APAFIS#7596). A schematic summary of the experimental protocol is presented in Fig. 1.

### Induction of chronic kidney disease

At 8 weeks of age, animals were randomly assigned to CKD or SHAM groups. CKD induction was performed as previously described^10^. Briefly, we applied cortical electrocautery to the right kidney through a 2-cm flank incision and then performed left total nephrectomy through a similar incision 2 weeks later. Control animals underwent SHAM operations. CKD and SHAM surgeries were performed by the same investigator for all mice.

### Transient middle cerebral artery occlusion (tMCAO)

Middle cerebral artery (MCA) occlusion was induced by the intraluminal filament method (for details, see Supplementary Methods) in both SHAM and CKD mice, 6 weeks after the last SHAM or CKD surgeries (i.e. in 16-week-old mice). Silicone rubber-coated monofilaments for MCAO model were obtained from Doccol Corporation (Sharon, USA). The diameter of the coated filaments was 0.21 +/− 0.02 mm (reference 6021910PK5Re). MCAO sutures in this category of coated tip diameter are suitable for MCAO models in animals within body weight range 22 +/− 2 grams. The induction of tMCAO has been performed by the same investigator but was not blinded regarding mice status (CKD or SHAM).

### Neurological evaluation

Neurological evaluations (neuroscore, rotarod test, prehensile test and grip test) were performed daily, 3 days before (d-3, d-2 and d-1) and 24 hours after stroke induction as described by Zausinger *et al*.^11^ (for details, see Supplementary Methods). No neurological evaluation was performed on the day of stroke induction (day 0). The scores obtained 24 hours before and 24 hours after stroke are presented in the manuscript.

### Hematology and serum biochemistry

Twenty four hours after MCAO, mice were deeply anaesthetized by intraperitoneal administration of ketamine (80 mg/kg) and xylazine (8 m/kg). Euthanasia was performed by total exsanguination. Blood samples were used for both hematology and serum biochemistry. Evaluation of hematologic parameters was performed on Genius KT 6200 VET system (Shenzhen Genius Electronics Co., LTD) following manufacturer’s instructions. Measurements of serum urea, phosphorus and calcium were performed on RX Daytona + system (Randox Laboratories).

### Brain preparation for histology

After exsanguination, mice were transcardially perfused with cold phosphate buffered saline (PBS), followed by 4% paraformaldehyde (PFA). Brains were removed and post-fixed for 24 additional hours in 4% PFA at 4 °C. After freezing in isopentane, the brain tissue was coronally sectioned on a cryostat at 12 levels according to stereotactic sections maps designed by K. Franklin and G. Paxinos^12^. Stereotaxic coordinates of the 12 regions of interest were expressed based on Bregma location (Supplementary Figs 1 and 2).

#### Measurement of infarct volume

To measure brain infarct volumes, a cresyl violet staining was performed for the 12 stereotactic regions (Supplementary Fig. 2B). The staining was performed on 30 µm sections to keep intact all brain structures. The unstained area of brain sections was defined as infarcted. Infarct volumes (cortical, striatal and total) were assessed by image analysis after digitization according to the method developed by Bordet *et al*.^13^ by image analysis software (Saisam®). Briefly, cortical and subcortical uncorrected infarcted areas and total hemispheric areas were calculated separately for each coronal slice. Total, cortical, and subcortical infarct volumes and hemispheric volumes (mm^3^) were calculated by the use of numerical integration of the respective areas for all the sections per animals and the distance between them. A corrected total infarct volume was calculated to compensate for the effect of brain edema. The corrected volume was calculated using the following equation: corrected infarct volume = infarct volume − (right hemisphere volume − left hemisphere volume).

#### Immunohistochemical examination of the ischemic area

All the immunohistological analyses were performed on the brain region displaying the coordinates: Bregma 0.00 mm. In this region, the ischemic core (striatum) is separated from the ischemic penumbra (cortex) by the corpus callosum, a structure that can be easily identified and allows differentiating the two zones (Supplementary Fig. 2B,C). Immunostainings were performed on brain sections of 20-μm using rabbit polyclonal IgG anti-NeuN, (Abcam ab104225, 1:500 dilution from original unit), goat polyclonal IgG anti-Iba1 (Abcam ab5076, 1:500 from original unit), rabbit polyclonal IgG anti-GFAP (Abcam ab7260, 1:500 dilution from original unit) as detailed in Supplementary Methods. For each animal the quantifications were performed on two different pictures of 90000 µm^2^ (2 pictures taken within the striatum and 2 pictures taken within the cortex). Each picture has been gridded and 3 fields of 10000 µm^2^ have been randomly selected. The number of positive cells per field was counted in the cortex and in the striatum. Data are presented as a number of positive cells/field. Quantifications were performed using the Histolab software (version 6.0.5, Microvision Instruments-Evry).

### TUNEL assay

*In Situ* Cell Death detection kit (Roche, cat. No. 11684817910) was used to detect individual apoptotic cells in brain frozen sections. A detailed protocol is given in Supplementary Methods. For each animal the quantification of the staining was performed on a single picture, taken at objective x5 (which represents a brain surface of 5 mm^2^, i.e around one quarter of the total brain surface of the section), using the Histolab software (version 6.0.5, Microvision Instruments-Evry). The whole picture of each animal has been gridded and 9 fields of 40000 µm^2^ have been randomly selected both in the striatum and in the cortex. The number of positive cells per field has then been counted in the cortex and in the striatum. Data are presented as a number of TUNEL positive cells/field of 40000 µm^2^.

### Real-time PCR

Total RNA from both the ischemic (ipsilateral) and the healthy (contralateral) hemispheres was isolated using mirVana^TM^ PARIS^TM^ Kit (Fisher Scientific, Illkirch, France) following the manufacturer’s instructions. First strand cDNAs were reverse transcribed from 1 µg of total RNA with the ‘High Capacity cDNA Reverse Transcription Kit’ (Applied Biosystems, Foster City, CA, USA) according to the manufacturer’s instructions. Real-time PCR reactions were performed in triplicate on StepOnePlus real-time PCR system (Applied Biosystems) using 2x SYBR green qPCR master mix (Applied Biosystems). The parameters for qPCR are presented in Supplementary Methods. The sequences of the PCR primers for each gene are presented in Supplementary Table 1. Levels of mRNA were normalized to the endogenous control, β-actin expression, and were calculated using fold change relative to the contralateral healthy hemisphere of the SHAM group.

### Western blot

Total proteins from ischemic (ipsilateral) hemispheres were isolated using mirVana^TM^ PARIS^TM^ Kit (Fisher Scientific, Illkirch, France) following the manufacturer’s instructions. Western blotting was performed using rabbit polyclonal anti-phosphorylated AMPK α1/2 (1:500, Santa Cruz Biotechnology, Santa Cruz, CA, USA), rabbit monoclonal anti-phosphorylated NFƘB p65 (1:1000, Cell Signaling Technology, Danvers, MA) or rabbit polyclonal anti-IƘBα (1:1000, Cell Signaling Technology, Danvers, MA) as detailed in Supplementary Methods.

### Statistical analyses

Results are expressed as the mean ± standard error of the mean (SEM). Data concerning serum biochemistry, hematology, ischemic volumes and immunohistochemistry were obtained from a first cohort of 14 SHAM-operated and 13 CKD mice. Concerning serum biochemistry, hematology, ischemic volumes and immunohistochemistry, differences between groups were analyzed using a non-parametric Mann-Whitney test. Western blot and real-time PCR analyses were performed on samples collected from a second cohort of 10 SHAM-operated and 12 CKD animals. Concerning real-time PCR, results for each group are expressed as a ratio compared to the mean value of expression measured in the contralateral hemisphere of SHAM-operated mice. Differences in mRNA levels between the contralateral and the ipsilateral hemispheres within each group were analyzed using the non-parametric Wilcoxon matched pairs test. A non-parametric Mann-Whitney test was performed to analyse differences in mRNA expression levels between SHAM-operated and CKD animals. Concerning western blot analyses, results are expressed as a ratio compared to the mean value of expression measured in the ipsilateral hemisphere of SHAM-operated mice. A non-parametric Mann-Whitney test was performed to analyse differences in protein expression levels between SHAM-operated and CKD animals. Data concerning weight loss and neurobehavioral impairments were obtained from both cohorts. Concerning weight loss, differences between groups were analyzed using a non-parametric Mann-Whitney test. Statistical analysis of neurobehavioral impairments were performed with a non-parametric Kruskall-Wallis test followed by Dunn’s multiple comparison posttests. The threshold for statistical significance was set at p ≤ 0.05. All statistical analyses were performed using Graphpad Prism software (Graphpad®). The design of the study did not allow the researchers to proceed to a blinded evaluation of the neurological impairments. However, for immunohistological and real-time PCR analyses, all samples were coded by an experimenter before to be processed and analyzed by another experimenter who was not aware of the coding.

## Results

### Body weight, serum biochemistry and hematology

Data on bodyweight as well as hematology and serum biochemistry parameters are presented in Supplementary Tables 2 and 3. Concentrations of urea, phosphorus and calcium were significantly higher in CKD-mice compared to SHAM-operated mice. Hematology parameters such as hemoglobin, red blood cell count, hematocrit, mean corpuscular volume and mean cell volume were significantly decreased in CKD-mice compared to SHAM-operated mice. No difference was observed in mean corpuscular hemoglobin concentration between both groups. Six weeks after SHAM or CKD surgeries, CKD mice displayed lower mean bodyweight compared with SHAM-operated mice. Post-stroke bodyweight loss was significantly higher in CKD- than in SHAM-operated mice.

### Uremia worsens infarct volume size and functional recovery in mice

**Figure 2:**
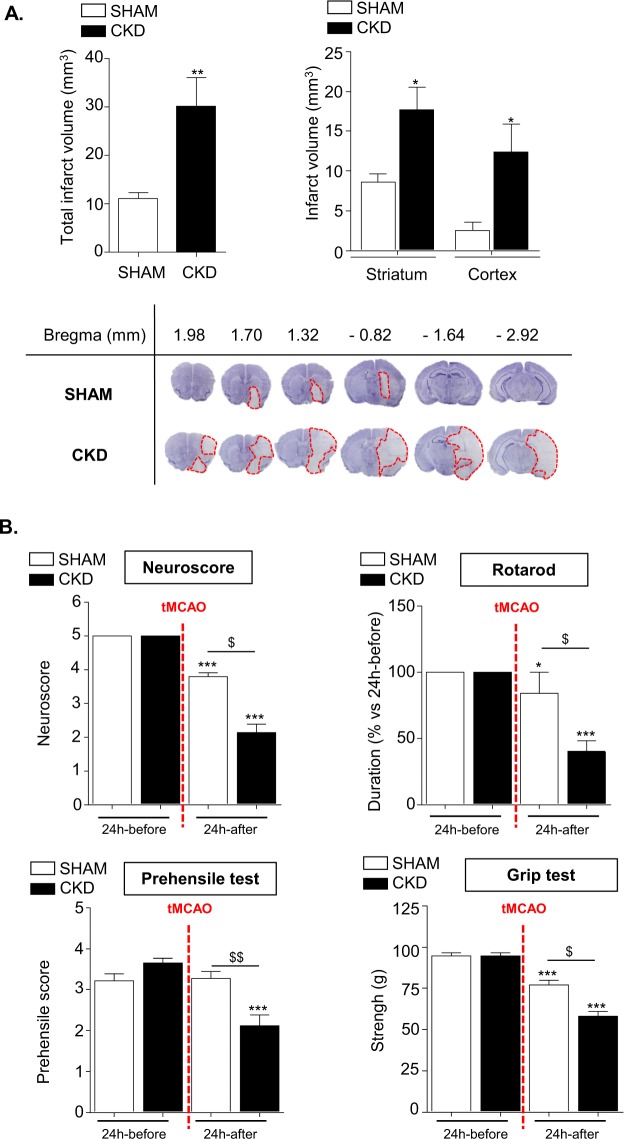
CKD worsens infarct volumes and impairs mice functional recovery after tMCAO. (**A**) Analysis of total (upper left) as well as cortical and striatal (upper right) infarct volumes in SHAM and CKD animals. The lower panels show representative pictures of cresyl-violet staining. Results are expressed as mean ± SEM. *p < 0.05 and **p < 0.01, CKD *versus* SHAM mice (non parametric Mann-Whitney U test). SHAM mice: n = 14. CKD mice: n = 13. (**B**) Neurobehaviourial evaluation performed on SHAM (n = 24) and CKD (n = 25) mice 24 hours before and 24 hours after tMCAO. Results are expressed as mean ± SEM. Statistical analysis was performed with a non-parametric Kruskall-Wallis test followed by Dunn’s multiple comparison posttests. *p < 0.05 24 hours before *versus* 24 hours after tMCAO within each group, ***p < 0.001 24 hours before *versus* 24 hours after tMCAO within each group; ^$^p < 0.05 CKD *versus* SHAM mice 24 hours after tMCAO, ^$$^p < 0.01 CKD *versus* SHAM mice 24 hours after tMCAO.

As described in Fig. 2A, the total volume of ischemic damage was significantly increased in CKD mice compared to SHAM-operated mice (32.9 ± 8.0 mm^3^ *vs* 12.8 ± 4.8 mm^3^). In SHAM-operated mice the infarcted area was mainly located within the striatum (ischemic core) whereas the volume of cortical infarction (ischemic penumbra) was limited. CKD mice displayed a significant increase in both striatal and cortical infarct volumes as compared to SHAM-operated mice (17.7 ± 2.8 mm^3^ vs 8,6 ± 1.1 mm^3^ and 12.4 ± 3.5 mm^3^ vs 2.5 ± 1.0 mm^3^ respectively, p < 0.05).

The neurological score, time spend on the rotarod, grip strength and prehensile abilities were similar in SHAM and CKD mice before stroke induction (Fig. 2B). After stroke induction, neurological score, time spend on the rotarod and grip strength were significantly decreased in both SHAM-operated and CKD mice and were significantly lower in CKD mice compared to SHAM-operated mice (p < 0.001). The prehensile score was similar in SHAM-operated mice before and after stroke. In contrast, the prehensile score was significantly decreased in CKD mice after stroke. This decrease was significant as compared to SHAM-operated mice (p < 0.001).

### Uremia amplifies stroke-induced apoptosis and neuronal loss

**Figure 3:**
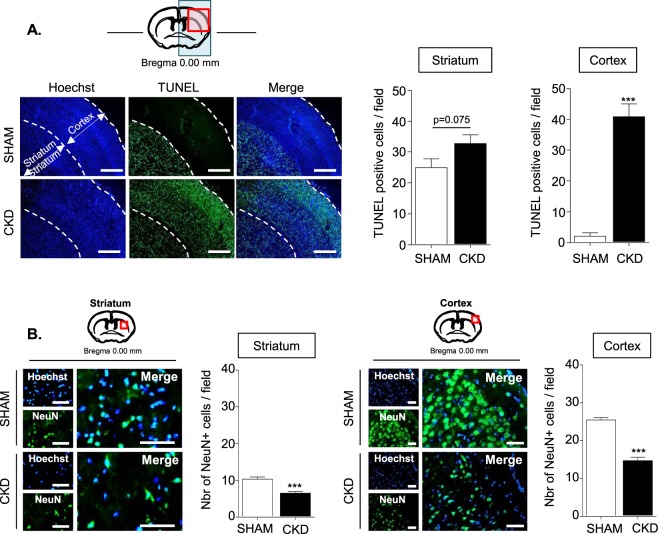
Both apoptosis and neuronal loss are increased in the cortex and the striatum of CKD mice. (**A**) The left panels show representative pictures of the TUNEL immunostaining performed in SHAM and CKD mice. Bars: 500 µm. Quantifications of TUNEL immunostaining in the striatum and the cortex are presented on the right side of the figure. Quantifications represent the number of TUNEL positive cells per fields of 40000 µm^2^. (**B**) Immunostaining analysis of NeuN expression in the striatum (left) and the cortex (right) of SHAM vs CKD mice. Bars: 50 µm. Quantifications represent the number of NeuN positive cells per fields of 10000 µm^2^. Results are expressed as mean ± SEM and represent data from at least 8 animals per group. ***p < 0.001 CKD *versus* SHAM mice (non parametric Mann-Whitney U test).

Apoptosis, evaluated by TUNEL staining, was observed in the striatum of both SHAM and CKD animals (Fig. 3A). While very few TUNEL positive nuclei were observed in the cortex from SHAM animals, a 40-fold increase in the surface of apoptosis was observed in the cortex of CKD animals. The number of TUNEL positive cells tended to increase in the striatum of CKD animals compared to SHAM animals, but the difference between both groups was not significant (p = 0.0751). A significant decrease of NeuN positive cells was observed both in the striatum and in the cortex of CKD mice compared to SHAM-operated mice (Fig. 3B).

### CKD impairs M2- and promoted M1-polarization of the microglia/macrophages in the ischemic brain

**Figure 4:**
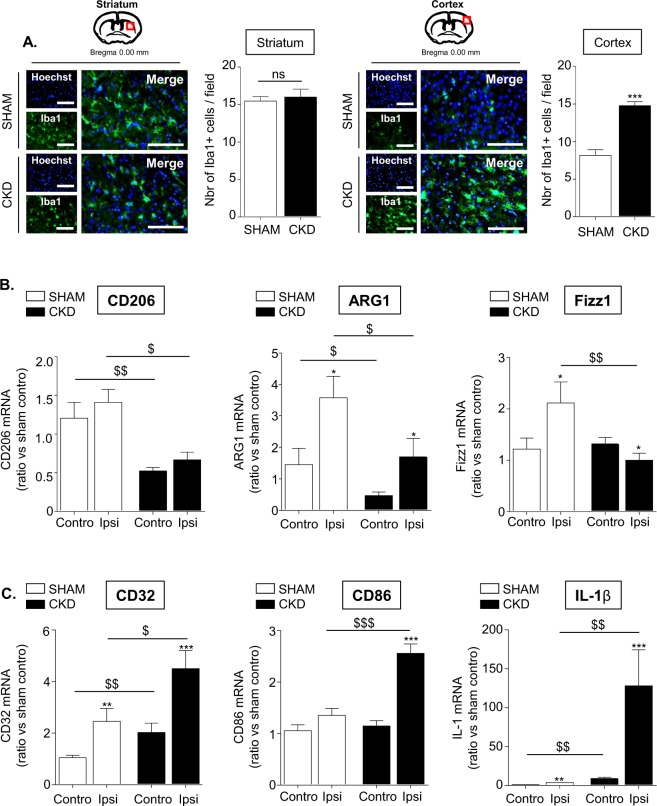
CKD promotes the recruitment of microglia/macrophages within the ischemic penumbra, impairs their M_2_- and promotes their M_1_-polarization. (**A**) Immunostaining analysis of Iba-1 expression (a marker of activated microglia) in the striatum (left) and the cortex (right) of SHAM vs CKD mice. Bars: 100 µm. Quantifications represent the number of Iba-1 positive cells per fields of 10000 µm^2^. Results are expressed as mean ± SEM and represent data from at least 8 animals per group. ***p < 0.001 CKD *versus* SHAM mice (non parametric Mann-Whitney U test). (**B**) Analysis by real-time PCR of the M_2_ markers CD206, ARG1 and Fizz1. (**C**) Analysis by real-time PCR of the M_1_ markers CD32, CD86 and IL-1β. Results are expressed as mean ± SEM and represent data from at least 8 animals per group. *p < 0.05, **p < 0.01, ***p < 0.001 ipsilateral *versus* contralateral hemisphere (non parametric Wilcoxon matched pairs test). ^$^p < 0.05, ^$$^p < 0.01, ^$$$^p < 0.001 CKD *versus* SHAM mice (non parametric Mann-Whitney U test). Contro: contralateral hemisphere; Ipsi: ipsilateral hemisphere.

Immuno-histological analyses of ionized calcium binding adapter molecule 1 (Iba1), a calcium-binding adapter protein which labels both monocytes and resting as well as activated microglia, had been performed to evaluate the recruitment of microglia/macrophages within ischemic lesions. Analyses of brain sections showed that the content in Iba1 positive cells in the striatum was equal between both groups (Fig. 4A). A significant increase in the number of cortical Iba1 positive cells was observed in CKD- compared to SHAM-operated animals (p < 0.001).

Using a real-time PCR approach, we observed that the M_2_ markers CD206, ARG1 and Fizz1 were significantly decreased within ischemic lesion of CKD mice compared to SHAM-operated animals (Fig. 4B, p < 0.05). In contrast, the expression of the M_1_ signature genes CD32, CD86 and IL-1β (Fig. 4C), as well as IL-6 and iNOS (Supplementary Fig. 3) was significantly increased within ischemic lesions of CKD mice compared to SHAM-operated mice 24 hours after tMCAO. No difference was observed for TNF-α mRNA expression between the ischemic hemispheres of CKD mice and SHAM-operated mice (Supplementary Fig. 3). Our data also show a significant increase in MCP1 (p < 0.05), ICAM-1 (p < 0.001), VCAM-1 (p < 0.001) and MMP3 (p < 0.05) mRNA expression in the ipsilateral hemispheres of CKD mice compared to SHAM-operated mice (Supplementary Fig. 4).

### AMP kinase phosphorylation is impaired in ischemic brain from CKD mice compared to SHAM-operated mice

**Figure 5:**
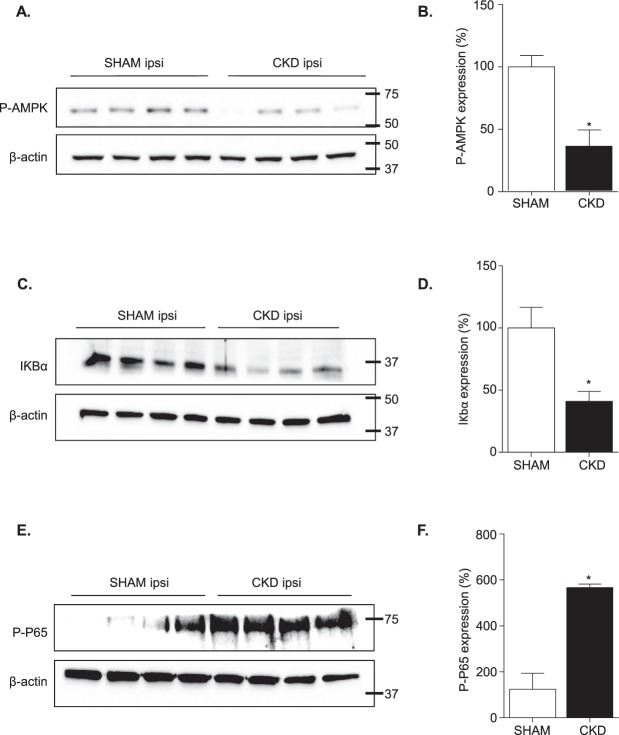
CKD impairs adenosine monophosphate-activated protein kinase (AMPK) activation and promotes canonical NFκB activation. (**A** and **B**) Study of AMPK activation. (**A**) Representative images of the western blot analysis. (**B**) Quantitative data showing lower AMPK phosphorylation within ischemic hemispheres of CKD mice as compared to SHAM-operated mice. (**C** and **D**) Study of IκBα degradation. (**C**) Representative images of the western blot analysis. (**D**) Quantitative data showing lower IκBα expression within ischemic hemispheres of CKD mice as compared to SHAM-operated mice. (**E** and **F**) Study of p65 phosphorylation. (**E**) Representative images of the western blot analysis. (**F**) Quantitative data showing higher phosphorylation of P65 within ischemic hemispheres of CKD mice as compared to SHAM-operated mice. Results are expressed as mean ± SEM and represent data from 4 animals per group. *p < 0.05 CKD *versus* SHAM mice (non parametric Mann-Whitney U test). Ipsi: ipsilateral hemisphere. Raw western blot data are provided in Supplementary Figs 5, 6 and 7.

Since adenosine monophosphate-activated protein kinase (AMPK) activation has been reported to promote macrophages/microglia polarization toward the M_2_ phenotype and to impair their M_1_ polarization^14,15^, we examined the impact of CKD on AMPK activation within ischemic brains. The western blot analysis demonstrated a significant decrease in AMPK phosphorylation within ischemic brain of CKD mice compared to SHAM-operated animals (Fig. 5A and B, p < 0.05). AMPK signaling was reported to impair M_1_ polarization in macrophages/microglia through the downregulation of NF-κB system^14,16^. Therefore, degradation of IκBα and phosphorylation of p65, which are hallmarks of NFκB pathway activation^17^, have been studied. The western blot analysis demonstrated a significant decrease of IKBα expression within ischemic lesions of CKD mice (Fig. 5C,D). This effect was associated with a significant increase of p65 phosphorylation (Fig. 5E,F). Raw western data is presented within Supplementary Figs 5, 6 and 7.

### Astrogliosis is amplified within the ischemic penumbra of CKD mice compared to SHAM-operated mice

**Figure 6:**
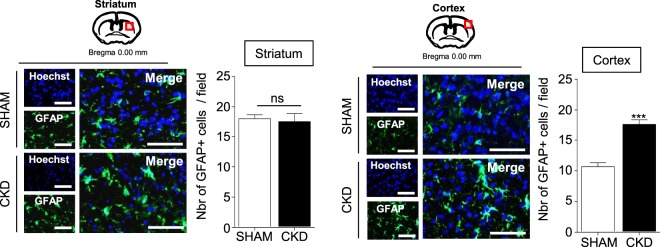
CKD amplifies the astrogliosis within the ischemic penumbra. Immunostaining analysis of GFAP expression (a marker of astrocytes) in the cortex (left) and the striatum (right) of SHAM vs CKD mice. Bars: 100 µm. Quantifications represent the number of GFAP positive cells per fields of 10000 µm^2^. Results are expressed as mean ± SEM and represent data from at least 8 animals per group. ***p < 0.001 CKD *versus* SHAM mice (non parametric Mann-Whitney U test).

Immuno-histological analyses of glial fibrillary acidic proteins (GFAPs), a marker of activated astrocytes, demonstrated a significant increase in the number of hypertrophic astrocytes in the ischemic cortex from CKD mice compared to SHAM-operated animals (Fig. 6, p < 0.001). The content of hypertrophic GFAP positive cells was similar in striatum from CKD- and SHAM-operated mice.

## Discussion

Our data demonstrate that in the acute phase of stroke recovery, CKD aggravates tMCAO-induced ischemic brain damage by increasing apoptosis and neuronal loss both in the ischemic core and the ischemic penumbra. As observed in humans^1,4^ the increased ischemic damage observed in mice are associated with impaired post-stroke recovery as evidenced by greater weight loss, decreased muscular strength, impaired motor coordination and reduced physical resistance to tiredness in CKD mice compared to SHAM-operated mice. Furthermore, our results indicate that the increased ischemic damage observed in CKD mice in the acute phase of stroke recovery is associated with an early increase in local inflammation due to increased M_1_ polarization of microglia/macrophages and an impairment of tissue reparation due to decreased M_2_ polarization.

CKD mice displayed lower mean bodyweight compared with SHAM-operated mice six weeks after SHAM or CKD surgeries (before tMCAO). However, there was no correlation between infarct volume size and mice bodyweight before tMCAO (r = 0.05385) (Supplementary Fig. 8A), suggesting that CKD-induced bodyweight loss had no influence on ischemic lesion severity and cannot be considered as a confounding factor for poor stroke recovery in this group. Interestingly, post-stroke bodyweight loss was significantly higher in CKD- than in SHAM-operated mice. The percent of weight loss after tMCAO significantly correlated with the total infarct volume (r = 0.6777; p = 0.0001) (Supplementary Fig. 8B) and a negative correlation was observed between post-stroke mice bodyweight and infarct volume size (r = −0.6660; p = 0.0001) (Supplementary Fig. 8C). This suggests that CKD-induced wider ischemic lesions were responsible for poorer recovery.

In the present work, we could not discriminate between microglia and macrophages polarization since all our attempts to identify monocytes/macrophages infiltration and polarization by immunohistochemistry failed. However, it is very likely that we observed the polarization of a mixed population of monocytes and microglia given the increased levels of MCP1 (involved in monocytes chemoattraction), ICAM-1 and VCAM-1 (involved in monocytes adhesion and rolling) and MMP3 (involved in blood-brain barrier disruption) observed within the ischemic hemispheres of CKD animals compared to SHAM animals (Supplementary Fig. 4). These data are in line with previous reports that showed increased ICAM-1 and VCAM-1 expression in response to elevated inorganic phosphate (Pi) in CKD animals^18^ and increased recruitment of leukocytes to the vascular wall in response to indoxyl sulfate (IS)^19,20^. For this reason, the term “microglia/macrophages” was used all along the manuscript.

Because reactive astrocytes secrete growth inhibitory molecules, reactive oxygen species (ROS), pro-inflammatory cytokines and MMPs, the astrogliosis that takes place after stroke occurrence is thought to worsen the ischemic damage^21,22^. In our model, the elevated number of astrocytes observed within the ischemic cortex of uremic mice displayed both elevated GFAP expression and hypertrophic morphology, suggesting that uremia may have amplified the reactive astrogliosis. In line with these data, uremic toxins (UTs) such as IS and quinolinic acid have been reported to promote the activation of astrocytes cultured *in vitro*, as evidenced by increased GFAP expression, together with iNOS expression, ROS production and the release of proinflammatory cytokines such as IL-1β and IL-6^23,24^. Therefore, the possibility that the astrogliosis observed in CKD animals may be linked to the accumulation of UTs cannot be ruled out and will be studied in the future.

Considering potential interventional therapies, according to our data, TNF-α is not involved in the aggravation of ischemic lesions induced by CKD and therefore does not represent an interesting therapeutic target in this context. AMPK recently emerged as a potential therapeutic target for ischemic stroke^25^. Among the main mechanisms involved, AMPK was reported to inhibit neuroinflammation, to decrease oxidative stress, to promote autophagy, to restrain glutamate release and excitotoxicity, to improve mitochondrial dysfunction and to inhibit apoptosis in ischemic stroke^25^. Interestingly, AMPK phosphorylation has been reported to impair M_1_ and favor M_2_ polarization of microglia/macrophages^14,15^. This effect appears to be mediated through AMPK-induced downregulation of NF-κB signaling^14,16^. In the present work, AMPK activation was reduced in ischemic brain from CKD animals compared to SHAM-operated animals. This effect was associated with decreased IκBα expression as well as increased p65 phosphorylation. Together these data suggest that CKD-induced M_1_ polarization and subsequent inflammation may be due to impaired AMPK signaling. These data are in accordance with a previous study, which reported that uremia increased the M_1_ and impaired the M_2_ polarization of macrophages through the inhibition of the AMPK^26^. Further studies will be needed to investigate whether the increased apoptosis observed within ischemic lesions of CKD mice is also linked to decreased AMPK activity.

In this study, microglia/macrophages recruitment and reactive astrocytes were increased exclusively within the ischemic penumbra of CKD mice, an area in which we observed a 40-fold increase in apoptosis and a 2-fold increase in neuronal loss. Interestingly, the ischemic penumbra is a tissue with a flow within the thresholds for maintenance of functional and morphologic integrity, which consequently has the potential for recovery^27^. The ischemic penumbra might therefore represent an interesting target for interventional therapies aiming to improve acute ischemic stroke recovery in this model.

Neurogenesis, which allows replacing damaged neurons, favors stroke recovery^28^. Ischemic hemispheres from CKD mice displayed reduced DCX expression as compared to SHAM-operated mice (Supplementary Fig. 9), suggesting that neurogenesis is impaired in uremic condition. In line with this observation, the accumulation in blood of the UT β−2 microglobulin (B2M) has been reported to promote age-related cognitive dysfunction and impaired neurogenesis in mice^29^. Therefore, the possibility that B2M-induced inhibition of neurogenesis may account for the decreased DCX observed in our model cannot be excluded. In the future it would be interesting to study whether the reduced neurogenesis observed 24 hours after tMCAO impacts long-term functional recovery in CKD animals. Arginase 1 plays a key role in neurogenesis. By degrading arginine, ARG1 favors the production of ornithine that leads to increased production of polyamines^30^, which stimulates neuronal progenitor proliferation^31^. In our model, ischemic hemispheres from CKD animals show decreased ARG1 expression. Further studies will be needed to determine whether CKD-induced decrease in ARG1 was responsible for impaired neurogenesis in this model.

As already mentioned, UTs represent potential therapeutic targets. Recently, UTs such as IS, p-cresyl sulfate and guanidino compounds were found to be highly expressed in uremic brains^32,33^. Evidence from *in vitro* studies suggests that these UTs may display direct deleterious effects within brain microenvironment. Indeed, molecules such as Pi and IS were reported to favor leukocyte adhesion to endothelial cells from large vessels and to promote cerebral endothelial cells dysfunction, through increased oxidative stress^8^. Exposure of macrophages/microglia^20,34–36^ and astrocytes^23,24^ to UTs amplified the release of inflammatory cytokines, oxidative stress and MMPs secretion. In addition, certain UTs, such as guanidino compounds, displayed direct neurotoxic properties *in vitro* due to excessive activation of the *N*-methyl-d-aspartate (NMDA) subtype of glutamate receptor^37,38^. Altogether, these mechanisms are thought to contribute to the poor stroke outcomes observed in CKD patients. However, to date, the lack of interventional approaches aiming to study ischemic brain lesions in animal models of CKD prevented firm conclusions to be drawn. In the future, the approach developed in the present work will be useful to investigate the impact of UTs on the severity of ischemic strokes *in vivo*.

### Limitations of the study

High blood pressure is frequently associated with renal failure and could play a role in ischemic stroke severity. Interestingly, the severity of the stroke can also be associated with a low blood pressure. Blood pressure before and after tMCAO have not been measured in this study, which represents a main limitation of the work. However, previous data from our group repeatedly reported that the induction of CKD in this mice model does not produce hypertension^39–41^. Therefore, it is unlikely that the impact of uremia on stroke recovery was linked to hypertension in this study. In addition, a recent study from Morris and colleagues reported that the cerebral blood flow, measured by laser Doppler in 8-week-old C57BL/6 J mice exposed to 15 minutes occlusion of the MCA by tMCAO, was not significantly altered after reperfusion, suggesting that the pressure is unlikely to be modified in this model^42^. Additional studies will be needed to ascertain that this parameter is not altered in our model.

In this study, experiments were performed only on female mice. Therefore, the possibility that the estrous cycle and/or gonadal hormones may have influenced stroke recovery cannot be ruled out. Future studies will be needed in order to assess the impact of CKD on stroke recovery on male mice.

## Summary/Conclusions

The *in vivo* approach used in this work successfully allowed us to study the mechanisms by which CKD aggravated ischemic stroke severity. We report that the ischemic penumbra may represent an interesting target for neuroprotective therapies in CKD patients. In addition, the secondary mediators of ischemic cell death and blood-brain barrier disruption, such as IL-6, IL-1β, MMP-3 and iNOS, may constitute useful biomarkers of CKD-induced stroke severity. The early increase of M_1_ polarization associated with uremia might play a central role in CKD-induced worsening of strokes outcomes. Therefore, interventional studies aiming to block microglia/macrophages M_1_ polarization, or directly target IL-6 and IL-1β, will be needed to evaluate if a causal link exists between inflammation and stroke severity in CKD. In this context, AMPK activity, which is known to block microglia/macrophages M_1_ polarization, and has been proved to be reduced in CKD, may represent an interesting therapeutic target. The possibility that uremic toxins such as IS, Pi and guanidino compounds, which display neurotoxic, inflammatory and pro-oxidative properties may be responsible for CKD-induced amplification of ischemic lesions cannot be excluded and will be tested in further studies by using pharmaceutical agents such as sevelamer or AST120.

## Supplementary information

Supplementary Information
