# Supplementary material for: Cellular and molecular mechanisms associated with ischemic stroke severity in female mice with chronic kidney disease
Source: Sci Rep. 2019 Apr 23;9:6432. doi: 10.1038/s41598-019-42933-0 (PMC6478694; doi:10.1038/s41598-019-42933-0)
Supplement: Supplementary file 1 — Supplementary Information [file 41598_2019_42933_MOESM1_ESM.pdf]

Supplementary Information

Cellular and molecular mechanisms associated with ischemic stroke severity in female mice with chronic kidney disease.

Authors: Hénaut Lucie[1#], PhD; Grissi Maria[1#], MSc; Brazier François[1,2], MD; Assem Maryam[1,2], MD; Poirot-Leclercq Sabrina[1]; Lenglet Gaëlle[1], PhD; Boudot Cédric[1], PhD; Avondo Carine[1]; Boullier Agnès[1,5], PhD; Choukroun Gabriel[1,2], MD, PhD ; Massy Ziad. A[3,4], MD, PhD; Kamel Saïd[1,5], PhD; Chillon Jean-Marc[1,6], PhD.

Authors' affiliations:

[1] MP3CV, EA7517, CURS, Jules Verne University of Picardie, Amiens, 80025 France

[2] Division of Nephrology, Amiens University Hospital, Amiens, 80054 France

[3] Division of Nephrology, Ambroise Paré University Hospital, Boulogne-Billancourt, 92104 France

[4] Inserm U1018, CESP Team 5, UVSQ, Villejuif, 94807 France

[5] Laboratory of Biochemistry, Amiens University Hospital, Amiens, 80054 France

[6] DRCI, Amiens University Hospital, Amiens, 80054 France

# These authors contributed equally to the work

Gene	Forward	Reverse
**iNOS**	CCC TTC AAT GGT TGG TAC ATG G	ACA TTG ATC TCC GTG ACA GCC
**TNFα**	ATG AGA AGT TCC CAA ATG GCC	CCA CTT GGT GGT TTG CTA CGA
**IL-1**	ACC TTC CAG GAT GAG GAC ATG A	CTA ATG GGA ACG TCA CAC ACC A
**IL-6**	GCT GGT GAC AAC CAC GGC CT	AGC CTC CGA CTT GTG AAG TGG T
**CD32**	AAT CCT GCC GTT CCT ACT GAT C	GTG TCA CCG TGT CTT CCT TGA G
**CD86**	GAG CGG GAT AGT AAC GCT GA	GGC TCT CAC TGC CTT CAC TC
**ICAM-1**	CCT GTT TCC TGC CTC TGA AG	GTC TGC TGA GAC CCC TCT TG
**VCAM-1**	CCC AAG GAT CCA GAG ATT CA	TAA GGT GAG GGT GGC ATT TC
**MCP1**	ATG CAG TTA ACG CCC CAC T	CAT TCC TTC TTG GGG TCA GC
**Fizz1**	ATG ACT GCT ACT GGG TGT GC	GCA GTG GTC CAG TCA ACG AG
**ARG I**	GAA CAC GGC AGT GGC TTT AAC	TGC TTA GCT CTG TCT GCT TTG G
**DCX**	AGC TGA CTC AGG TAA CGA CCA	GCT TTG ACT TAG GTG TTG AGA GC
**MMP9**	ATG GTG CCC CAT GTC ACT TT	AAC GGG AAC ACA CAG GGT TT
**MMP2**	TCT GAT GGC CCC GAT CTA CA	CAG TAT CAG CAT CGG GGG AG
**MMP3**	GTC CTC CAC AGA CTT GTC CC	GTA CCA CGA GGA CAT CAG GG

Supplementary Table 1: qRT-PCR primers sequences.

	SHAM (n=24)	CKD (n=25)	p value
**Body weight**			
Before tMCAO	21.70<br>(19.20 - 24.95) [20.55 - 22.20]	20.53<br>(17.50 – 22.80) [19.33 - 21.90]	p = 0.0219
After tMCAO	18.50<br>(17.00 – 23.10) [18.10 – 19.45]	16.90<br>(15.00 – 19.50) [15.70 – 17.50]	p < 0.0001
% of weight loss	11.86<br>(4.83 – 19.63) [9.048 – 16.10]	16.31<br>(5.79 – 32.13) [11.90 – 23.18]	p = 0.0054

Supplementary Table 2: Effects of CKD on body weight before and after tMCAO. Abbreviations: CKD: chronic kidney disease; tMCAO: transient middle cerebral artery occlusion. Data are expressed as median value. The min and max value are presented in parentheses. Interquartile values are written in square brackets.

	Reference values for mice	SHAM (n=14)	CKD (n=13)	p value
**Serum Biochemistry**				
Urea (mmol/L)	2.85 – 11.78	7.99<br>(5.92 – 10.10) [7.17 – 8.52]	21.37<br>(17,76 – 25.22) [18.67 – 23.23]	$p < 0.001$
BUN (mg/dL)	10 – 33	22.38<br>(16.58 – 28.29) [20.10 – 23.88]	59.86<br>(49.75 – 70.64) [52.29 – 65.07]	$p < 0.0001$
Phosphorous (mmol/L)	1.98 – 3.01	2.81<br>(2.18 – 3.86) [2.63 – 3.36]	3.96<br>(2.26 – 5.24) [3.26 – 4.54]	$p = 0.0051$
Calcium (mmol/L)	1.77 - 2.52	3.38<br>(2.28 – 2.80) [2.36 – 2.46]	2.72<br>(2.54 – 3.6) [2.61 – 2.86]	$p < 0.001$
**Hematology**				
Hemoglobin (g/dL)	10 - 20	12.50<br>(9.80 – 14.60) [11.63 – 13.08]	9.8<br>(4.2 – 11.70) [9.15 – 10.40]	$p < 0.0001$
Red blood cells ($x10^{12}$/L)	7 - 11	6.78<br>(5.38 – 7.82) [6.51 – 6.94]	5.47<br>(2.36 – 6.25) [5.07 – 5.71]	$p < 0.0001$
Hematocrit (%)	35 - 40	40.20<br>(31.95 – 46.20) [38.45 – 41.73]	31.20<br>(26.20 – 34.90) [29.70 – 32.65]	$p < 0.0001$
MCV (fL)	45.5 - 60.3	59.45<br>(58.70 – 61.70) [59.08 – 59.70]	56.76<br>(56.00 – 58.30) [56.10 – 57.20]	$p < 0.0001$
MCH (pg)	14.1 - 19.3	18.05<br>(17.00 – 20.40) [17.43 – 18.80]	17.80<br>(15.90 – 18.60) [16.85 – 18.60]	$p = 0.1586$
MCHC (g/dL)	30.2 - 34.2	30.15<br>(28.10 – 33.90) [29.13 – 31.08]	31.30<br>(26.10 – 32.80) [29.38 – 32.10]	$p = 0.1027$

Supplementary Table 3: Effects of CKD on routine serum biochemistry and hematology parameters. Abbreviations: CKD: chronic kidney disease; MCH: mean corpuscular hemoglobin; MCHC: mean corpuscular hemoglobin concentration; MCV: mean corpuscular volume; tMCAO: transient middle cerebral artery occlusion. Data are expressed as median value. The min and max value are presented in parentheses. Interquartile values are written in square brackets.

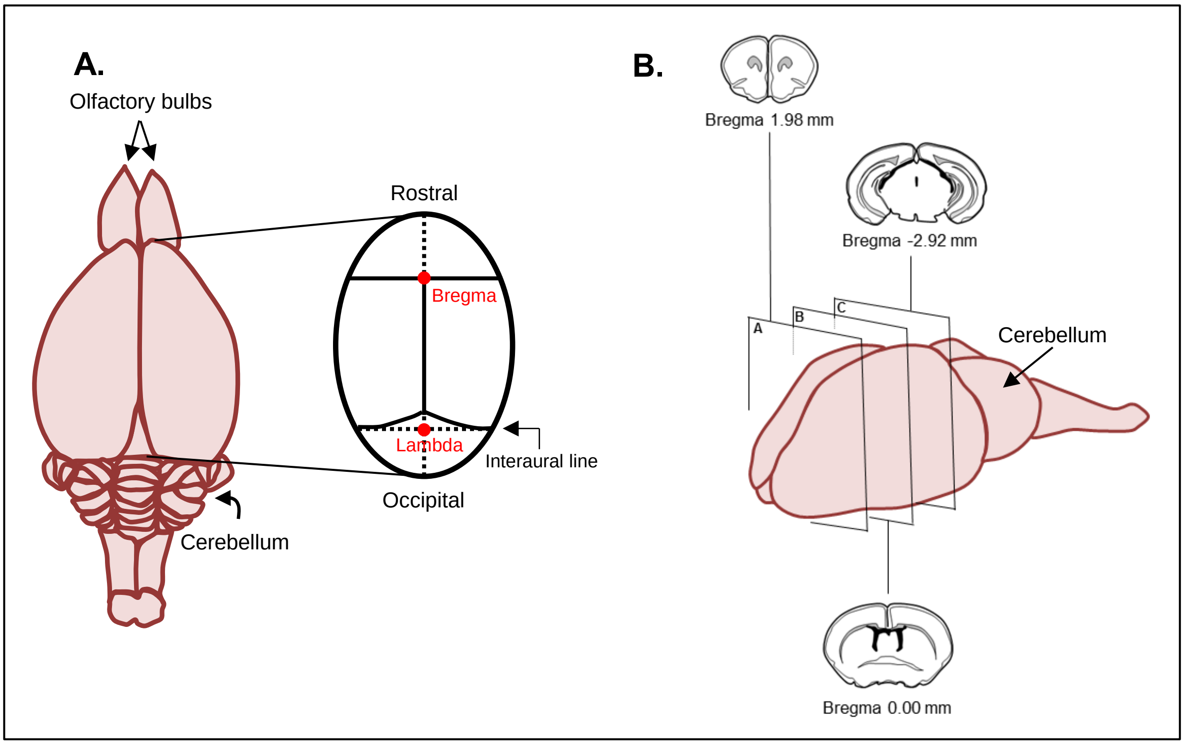

Supplementary Figure 1: Construction of the stereotaxic atlas of mouse brain. A stereotaxic atlas is a 3D reconstruction of the brain compiled from serial sections and drawings of sectioned brains. The Atlas is constructed such that as you move from a section to another you are travelling through the brain. Most of the available stereotaxic atlases of mice brain have coordinate systems for reaching brain structures stereotaxic localization. Stereotaxic coordinate system does not have a single universally accepted reference point. In the mice the most common reference points are bregma, lambda and the interaural line (IAL) In this study, Bregma was used as the zero reference. **A.** Stereotaxic atlases coordinate system. Bregma and Lambda are intersections of bone plates on the dorsal skull surface. The diagram indicates the approximate position of the interaural line, but this reference point is not located anatomically. **B.** Example of stereotaxic regions location using bregma as a zero reference. Commonly, brain structures located between the olfactory bulbs and the bregma (i.e before the zero reference) display positive coordinates. These coordinates represent the distance in millimeter between brain structures (located in the rostral side) and the bregma.

This means that the more a structure is close to the olfactory bulbs, the greater is its distance in millimeter from the bregma. Conversely, brain structures located between the bregma and the cerebellum display negative bregma coordinates (they are located behind the zero reference).

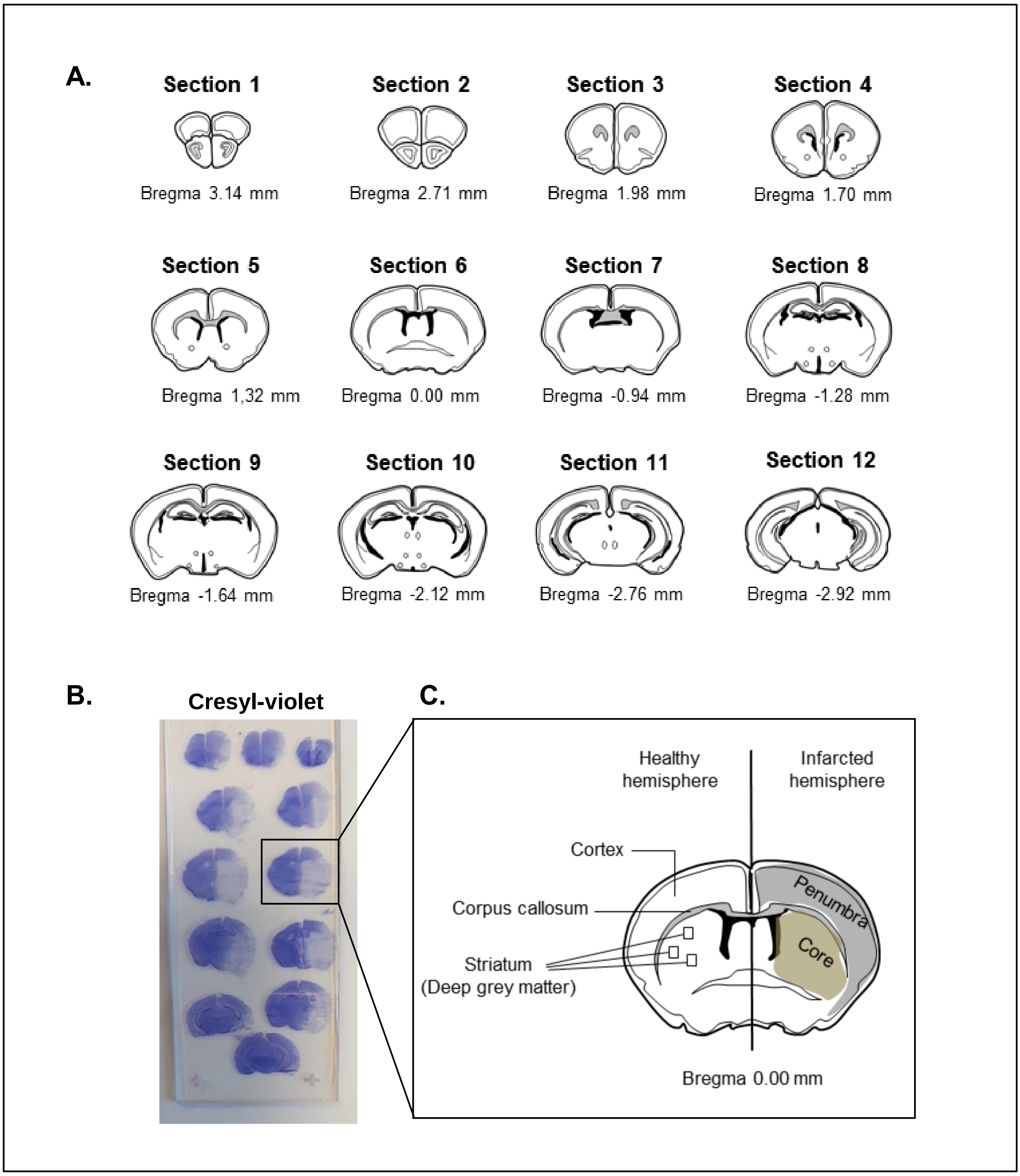

<u>Supplementary Figure 2:</u> Stereotaxic regions used to quantify brain ischemic volumes. A. Coordinates of the 12 stereotaxic sections used to quantify brain ischemic volumes. This schematic representation has been created based on K. Franklin and G. Paxinos stereotaxic atlas (The Mouse Brain in Stereotaxic Coordinates, Academic Press, 2012). **B.** Examples of a microscope slide stained with cresyl-violet. It is well established that 2 to 6 hours after tMCAO, the histological infarct core is limited to the striatum. Subsequently the core expands

to involve most of the cortical tissue supplied by the middle cerebral artery. This occurs quite rapidly and is complete within 6–12 hours after stroke onset. **C.** Description of section 6. All the immunohistological analyses were performed on the brain region displaying the coordinates: Bregma 0.00 mm (section 6). In this region, the ischemic core (striatum) is separated from the ischemic penumbra (cortex) by the corpus callosum, a structure that can be easily identified and allows to differentiate the two zones.

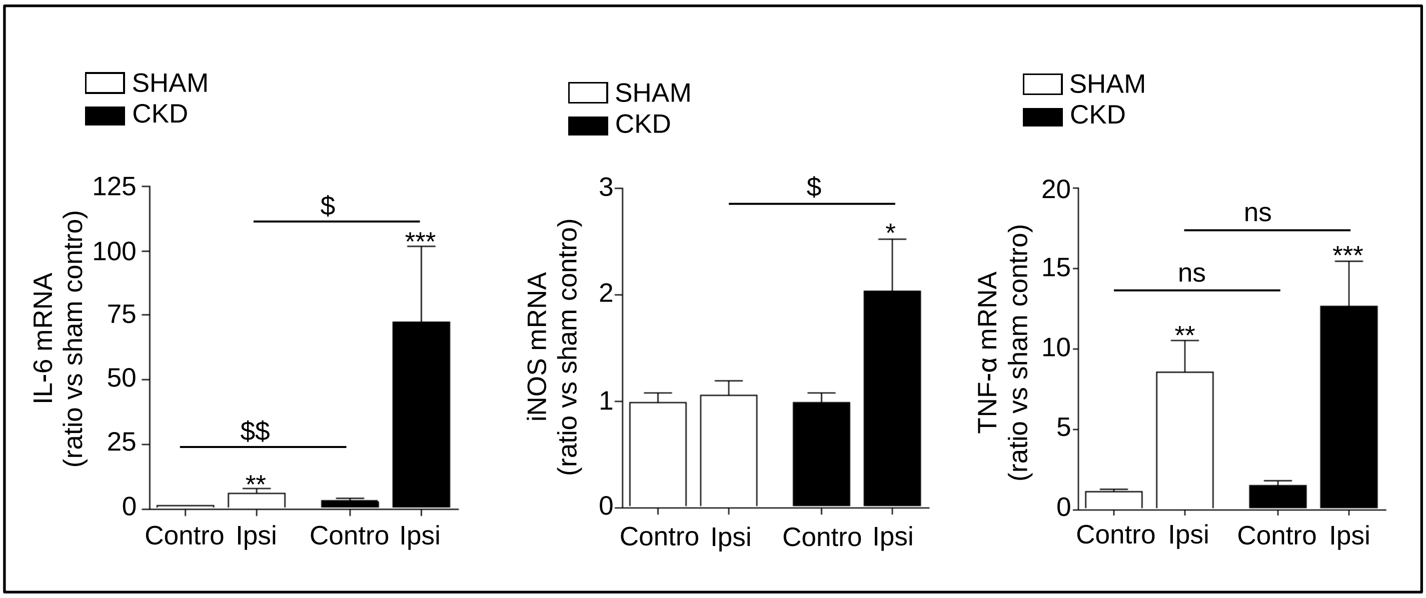

<u>Supplementary Figure 3:</u> Analysis by qRT-PCR of the M_1 markers IL-6, iNOS and TNF-α. Results are expressed as mean ± SEM and represent data from at least 8 animals per group. *: $p<0.05$, **: $p<0.01$, ***: $p<0.001$ ipsilateral *versus* contralateral hemisphere (non parametric Wilcoxon matched pairs test). $: $p<0.05$, $$: $p<0.01$, $$$: $p<0.001$ CKD *versus* SHAM mice (non parametric Mann-Whitney U test). Contro: contralateral hemisphere; Ipsi: ipsilateral hemisphere.

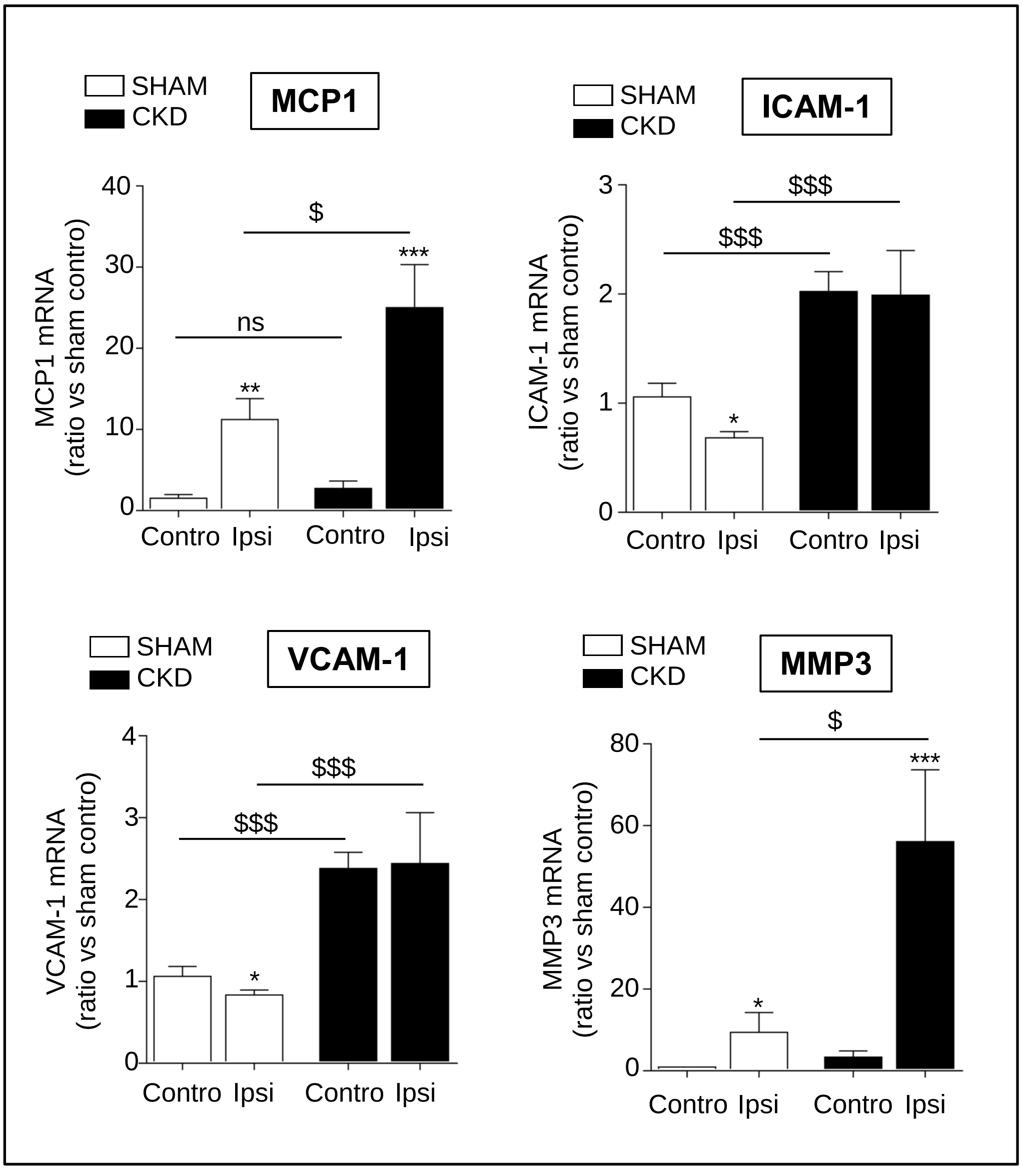

<u>Supplementary Figure 4:</u> Analysis by real-time PCR of MCP1, ICAM-1, VCAM-1 and MMP3 mRNA expression. Results are expressed as mean ± SEM and represent data from at least 8 animals per group. *: $p<0.05$, **: $p<0.01$, ***: $p<0.001$ ipsilateral *versus* contralateral hemisphere (non parametric Wilcoxon matched pairs test). $: $p<0.05$, $$$: $p<0.001$ CKD *versus* SHAM mice (non parametric Mann-Whitney U test). Contro: contralateral hemisphere; Ipsi: ipsilateral hemisphere.

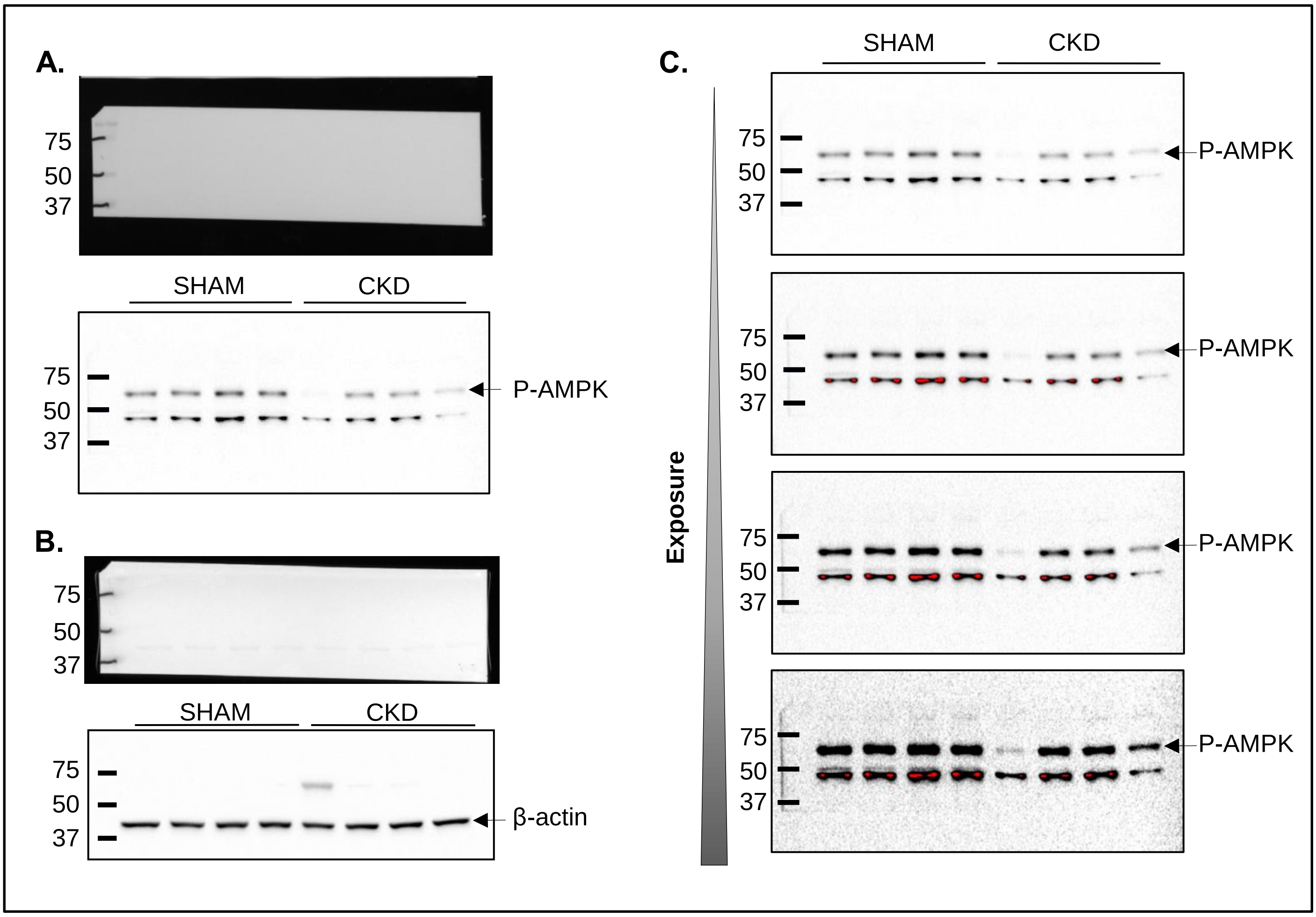

<u>Supplementary Figure 5:</u> CKD impairs adenosine monophosphate-activated protein kinase (AMPK) activation: raw western blot data. A. Full-length blot showing decreased AMPK phosphorylation in CKD animals. **B.** Full length blot showing β-actin expression. **C.** Multiple exposures of AMPK phosphorylation full-length blot showing decreased AMPK phosphorylation in CKD animals.

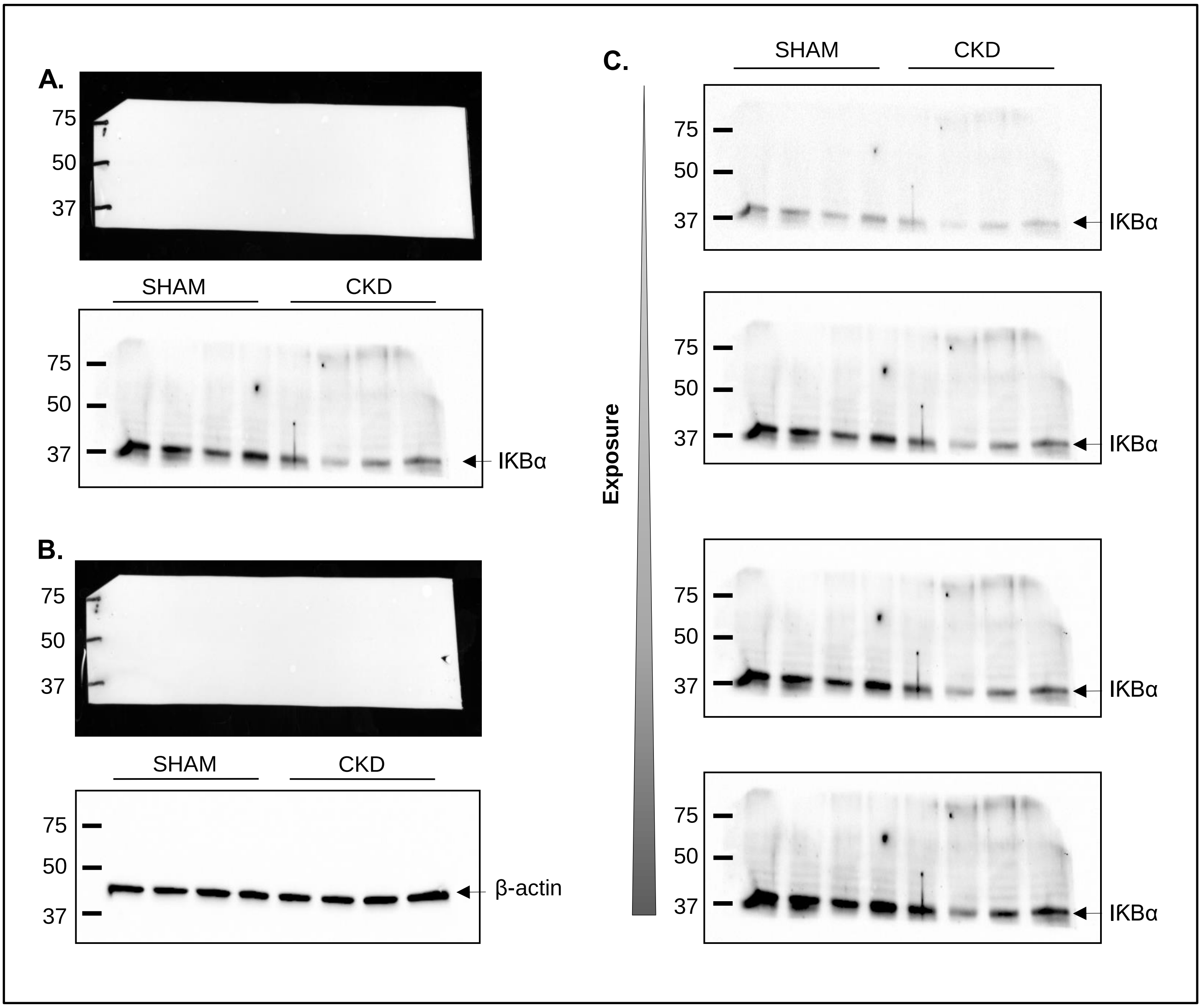

<u>Supplementary Figure 6:</u> CKD promotes IҡBα degradation: raw western blot data. A. Full-length blot showing decreased IҡBα expression in CKD animals. **B.** Full length blot showing β-actin expression. **C.** Multiple exposures of IҡBα full-length blot showing decreased IҡBα expression in CKD animals.

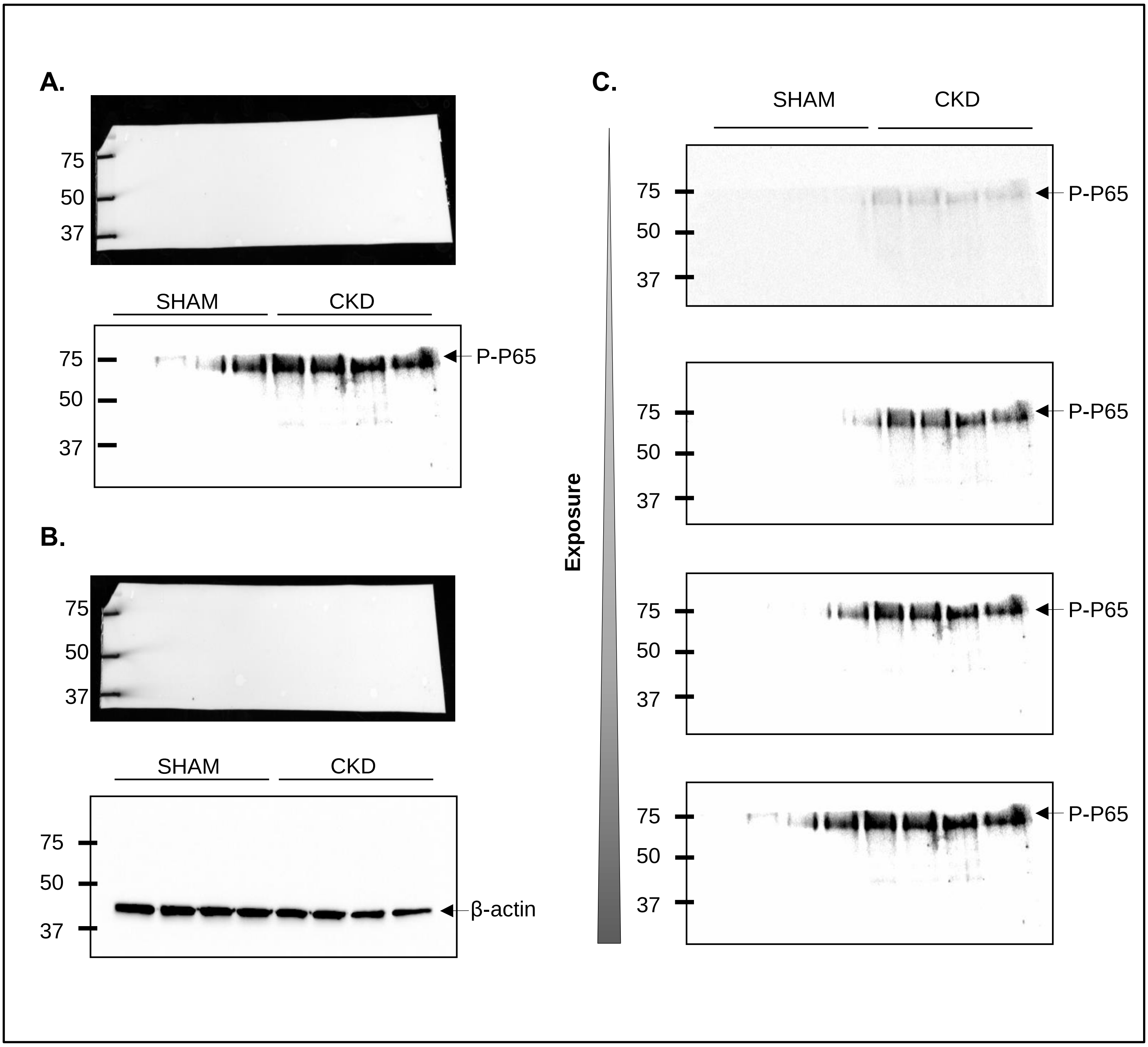

<u>Supplementary Figure 7:</u> **CKD promotes P65 phosphorylation within ischemic lesions: raw western blot data. A.** Full-length blot showing increased P65 phosphorylation in CKD animals. **B.** Full length blot showing β-actin expression. **C.** Multiple exposures of P65 phosphorylation full-length blot showing increased P65 phosphorylation in CKD animals.

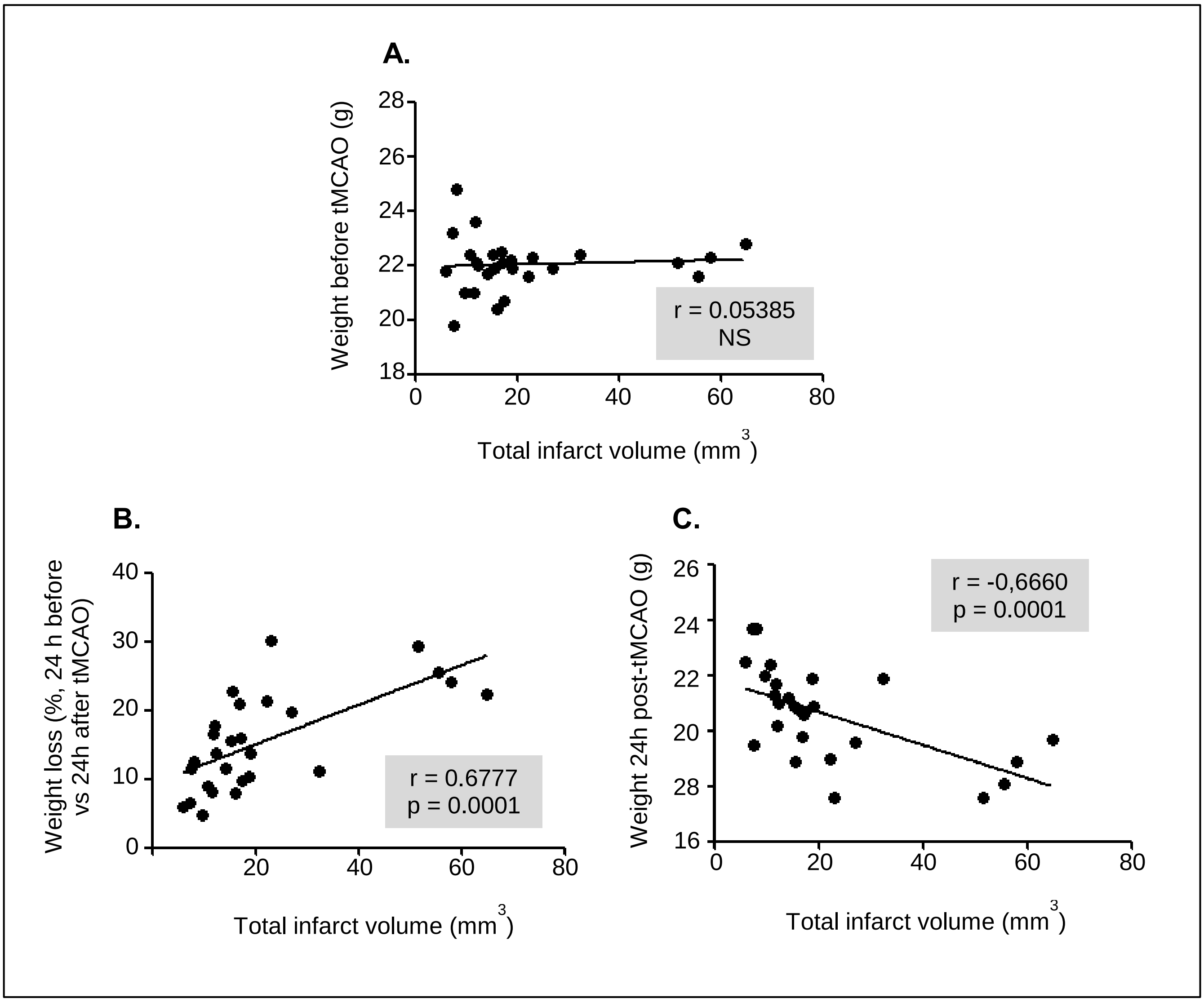

<u>**Supplementary Figure 8:**</u> **Correlations between mice bodyweight and brain ischemic volume. A.** Correlation between mice weight before tMCAO and total infarct volume. **B.** Correlation between the percent of weight loss (before vs after tMCAO) and brain total infarct volume **C.** Correlation between mice weight after tMCAO and total infarct volume. Statistical analysis was performed with a non-parametric Spearman correlation test. n = 27 mice (14 SHAM / 13 CKD).

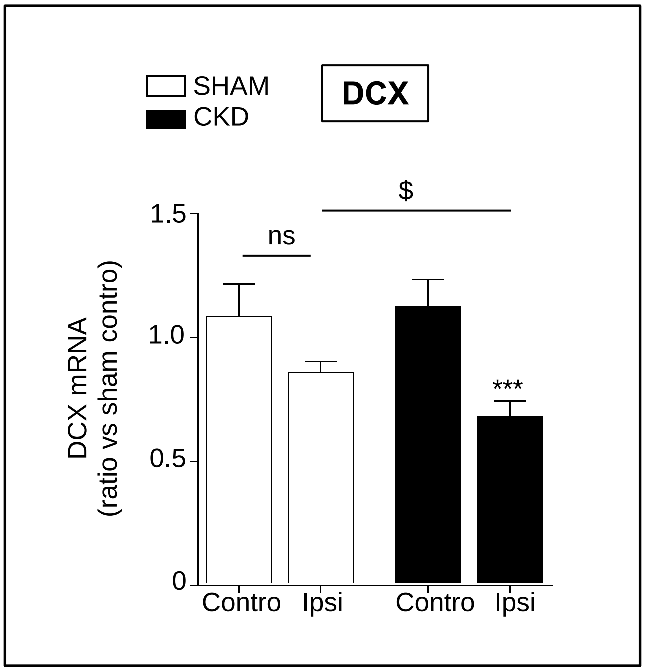

<u>Supplementary figure 9:</u> Neurogenesis is reduced in ischemic lesions of CKD mice. Analysis by qRT-PCR of the mRNA expression of the immature neuronal marker DCX. Results are expressed as mean ± SEM and represent data from at least 8 animals per group. ***: $p<0.001$ ipsilateral *versus* contralateral hemisphere (non parametric Wilcoxon matched pairs test). $: $p<0.05$ CKD *versus* SHAM mice (non parametric Mann-Whitney U test). Contro: contralateral hemisphere; Ipsi: ipsilateral hemisphere.

Supplementary Methods

Transient middle cerebral artery occlusion (tMCAO)

Ischemic lesions were induced in both SHAM and CKD mice, 6 weeks after the last SHAM or CKD surgeries (i.e. in 16-week-old mice). Briefly, mice were anesthetized with ketamine (80 mg/kg) plus xylazine (8 mg/kg) and a 20-mm-long 6-0 silicon rubber–coated nylon monofilament (Doccol®, Sharon, Massachusetts, USA) was inserted into the right common carotid artery. The filament was then advanced in the internal carotid artery and passed into the intracranial circulation (12–13 mm distal to the carotid bifurcation), thereby occluding the origin of the MCA. The right MCA was occluded for 15 minutes. The filament was then removed carefully to produce the reperfusion. Animals were allowed to recover for 24 hours and then euthanized for infarct volume analysis.

Neurological evaluation

- Neuroscore: A six-grade neuroscore was used to assess post-ischemic motor and behavioral impairments. Mice were graded as follows. Grade 5: mice were held gently by the tail, suspended one meter above the ground and observed for forelimb flexion. Normal mice extended both forelimbs toward the floor. Mice that extended both forelimbs toward the floor and did not display other neurological impairments were assigned a grade of 5. Grade 4: mice with consistent flexion of the forelimb contralateral to the injured hemisphere (varying from mild wrist flexion and shoulder adduction to severe posturing with full flexion of wrist and elbow, and induction of the shoulder with internal rotation) were assigned a grade of 4. Grade 3: mice were placed on a large sheet of soft, plastic-coated paper that they could grip firmly with their claws. The experimenter held the mouse by the tail and applied gentle lateral pressure at the animal's shoulder until the forelimbs slid several centimeters. The maneuver

was repeated several times to the left and to the right. Normal mice and slightly impaired mice resisted sliding to an equivalent extent in each direction. However, severely impaired mice with consistently reduced resistance to pushing towards the paretic side were assigned a grade of 3. Grade 2: mice were then allowed to move about freely and were observed for circling behavior when their tail was pulled. Mice that circled consistently towards the paretic side were assigned a grade of 2. Grade 1: mice were allowed to move about freely and were observed for circling behavior. Mice that circled spontaneously and consistently toward the paretic side were assigned a grade 1. Grade 0: mice without any spontaneous motion were assigned a grade of 0.

- Rotarod test: Motor coordination and physical resistance to tiredness were evaluated by using an accelerating rotarod. Mice were placed on a rotating horizontal cylinder. The rotation speed was increased from 5 revolutions per minute to a maximum of 20 revolutions per minute over a 2-minute period. The duration for which the mouse remained on the device (in seconds) was measured. The rotarod test was performed three times per session for up to 5 minutes and the best time on the cylinder was retained.

- Prehensile test: A prehensile test was performed by using a horizontal stainless steel wire (length: 60 cm; diameter: 3 mm) placed 40 cm above a foam pad. The mouse's forepaws were placed onto the wire and the animal was released. The time until the animal fell and the animal's ability to grab the wire with a hind paw were measured. The tested animals were scored as follows: 3 points for holding onto the wire for more than 10 seconds; 2 points for holding on for between 5 and 10 seconds on the wire; 1 point for holding on for between 1 and 5 seconds; 0 point for not being able to hang on. An additional point was added if the animal managed to grab the wire with a hind paw.

- Grip-test: The muscular strength of mice forelimbs was assessed using a grip strength test (Bioseb, Vitrolles, France). The grip strength meter was positioned horizontally and the mice

were held by the tail and lowered towards the apparatus. The animals were allowed to grab the metal grid and were then pulled backwards in the horizontal plane. The force applied to the grid just before the animals lost grip was recorded as the peak tension. The muscular strength of mice forelimbs was assessed 5 times per session and the mean of the 5 measurements was used for evaluation.

Immunohistochemical examination of the ischemic area

The sections dedicated to immuno-histology were cut with a thickness of 20 µm, i.e. thinner than sections dedicated to cresyl violet staining, in order to facilitate antibodies penetration within brain tissues. Sections were fixed with 4% ice-cold PFA for 5 min at room temperature (RT) and incubated in sodium citrate (1M, pH=6) during 20 min at 100°C for antigen retrieval. Section were then quenched in 100 mmol/L glycin in PBS for additional 10 min and permeabilized for 1h at RT with 0.3% triton X-100 in PBS containing 1% Bovine Serum Albumin (BSA). Non-specific binding of the antibody was blocked by incubation in a blocking solution (1% BSA in PBS) for 30 min at RT. Sections were then incubated overnight at 4°C with primary antibody (rabbit polyclonal IgG anti-NeuN, Abcam ab104225, 1:500 dilution from original unit; goat polyclonal IgG anti-Iba1, Abcam ab5076, 1:500 from original unit; rabbit polyclonal IgG anti-GFAP, Abcam ab7260, 1:500 dilution from original unit) prepared in PBS containing 0.3% triton X-100 and 1% BSA. Brain sections were then rinsed and incubated with secondary antibody (Alexafluor® 488 goat anti-rabbit IgG, Invitrogen A11008; Alexafluor® 488 goat anti-rat IgG, Invitrogen A11004; Alexafluor® 488 goat anti-goat IgG, Invitrogen A11055; 1:500 dilution from original unit), prepared in PBS 1% BSA, for 1 h at RT. Samples were then widely washed in PBS. Nuclei were counterstained with Hoechst and samples were mounted with Mowiol solution (Mowiol® 4-88, Sigma-Aldrich, St. Quentin Fallavier, France) for fluorescent detection.

TUNEL assay

Briefly, sections of 20-µm were fixed with 4% ice-cold PFA for 5 min at RT and incubated in sodium citrate (1M, pH=6) during 20min at 100°C for antigen retrieval. Section were then quenched in 100 mmol/L glycin in PBS for additional 10 min and permeabilized for 1h at RT with 0.3% triton X-100 in PBS containing 1% BSA. Non-specific binding of the antibody was blocked by incubation in a blocking solution (1% BSA in PBS) for 30 min at RT. Samples were then incubated for 60 min at 37°C in the TUNEL reaction mixture (prepared according to manufacturer's instructions), in a humidified atmosphere in the dark. Nuclei were counterstained with Hoechst and samples were mounted with Mowiol solution (Mowiol® 4-88, Sigma-Aldrich, St. Quentin Fallavier, France) for fluorescent detection.

Real-time PCR

The parameters for qPCR were as follows: a pre-amplification was performed with the following touchdown PCR protocol: 95°C for 10 min, 95°c for 15 sec, 66°C for 1 min, 95°C for 15 sec, 64°C for 1 min, 95°C for 15 sec, 62°C for 1 min; followed by 40 cycles of 95°C for 15 s, and 60°C for 1 min. The primers sequences used are listed in supplementary Table 1.

Western blot

Western blot was performed to examine AMP-activated protein kinase (AMPK) phosphorylation in the ischemic hemisphere of SHAM and CKD mice. Total proteins from ischemic (ipsilateral) hemispheres were isolated using mirVana™ PARIS™ Kit (Fisher Scientific, Illkirch, France) following the manufacturer's instructions. Samples were then separated by 10–12% SDS-PAGE under reducing conditions. After electrophoresis, samples were transferred onto nitrocellulose membranes, blocked with 5% milk in PBS containing

0.5% Tween 20 (PBS/Tween) for 1 h, washed with PBS/Tween and incubated overnight at 4 °C with rabbit polyclonal anti-phosphorylated AMPK α1/2 (1: 500, Santa Cruz Biotechnology, Santa Cruz, CA, USA), rabbit monoclonal anti-phosphorylated NFƘB p65 (1:1000, Cell Signaling Technology, Danvers, MA), rabbit polyclonal anti-IƘBα (1:1000, Cell Signaling Technology, Danvers, MA). Antibodies were diluted in 5% milk PBS/Tween. Blots were then washed with PBS/Tween and incubated with appropriate horseradish peroxidase-conjugated secondary antibody (1 : 5000, Santa Cruz Biotechnology, Santa Cruz, CA, USA). After washing with PBS/Tween, blots were developed with the chemiluminescence method (ECL) (Amersham, Aylesbury, UK) and then probed with mouse monoclonal anti-β-actin antibody (1 : 5000, Sigma-Aldrich, St. Quentin Fallavier, France) for control loading.
